# Injectable hydrogels for the sustained delivery of a HER2-targeted antibody for preventing local relapse of HER2+ breast cancer after breast-conserving surgery

**DOI:** 10.7150/thno.36514

**Published:** 2019-08-14

**Authors:** Xiaobin Chen, Maoli Wang, Xiaowei Yang, Yaoben Wang, Lin Yu, Jian Sun, Jiandong Ding

**Affiliations:** 1 State Key Laboratory of Molecular Engineering of Polymers, Department of Macromolecular Science, Fudan University, Shanghai 200438, China; 2 Department of Breast Surgery, Obstetrics and Gynecology Hospital, Fudan University, Shanghai 200011, China

**Keywords:** Injectable hydrogel, Breast-conserving surgery, Herceptin, Cardiotoxicity, Anti-relapse, Immunotherapy

## Abstract

A high risk of local relapse is the main challenge of HER2+ breast cancer after breast-conserving surgery. We aimed to develop a long-acting delivery system for Herceptin, a HER2-targeting antibody, using injectable and thermosensitive hydrogels as the carrier to prevent the local relapse of HER2+ breast tumors while minimizing systemic side effects, especially cardiotoxicity.

**Methods**: Two poly(lactic acid-*co*-glycolic acid)-*b*-poly(ethylene glycol)-*b*-poly(lactic acid-*co*-glycolic acid) (PLGA-PEG-PLGA) triblock copolymers with different PEG/PLGA proportions were synthesized. Their mixtures with rational mix proportions displayed sol-gel transitions in water with rising of temperature and the Herceptin-loaded hydrogel systems were then prepared. Both the *in vivo* antitumor and anti-relapse efficacies were evaluated after hypodermic injection of the Herceptin-loaded hydrogel, and the cardiotoxicity was also detected.

**Results**: The gel performance, degradation rate and drug release kinetics of hydrogels were easily adjustable by simply varying the mix proportion. The hydrogel matrix with a specific mix proportion not only avoided initial burst release but also achieved sustained release of Herceptin *in vitro* for up to 80 days, which is the longest period of Herceptin delivery that has ever been reported. *In vivo* biodistribution studies performed in SK-BR-3 tumor-bearing mice revealed that a single hypodermic administration of the Herceptin-loaded hydrogel adjacent to the tumor tissue promoted the intratumoral antibody accumulation. This resulted in a better antitumor efficacy compared to weekly hypodermic injections of Herceptin solution for 28 days. A tumor relapse model was also established by imitative breast-conserving surgery on tumor-bearing mice, and both the single injection of the Herceptin-loaded hydrogel and the weekly injection of the Herceptin solution achieved superior anti-relapse efficacy. Furthermore, both antitumor and anti-relapse experiments demonstrated that the weekly pulsed administration of the Herceptin solution caused cardiotoxicity; however, the sustained release of Herceptin from the hydrogel effectively prevented this side effect.

**Conclusion**: The Herceptin-loaded hydrogel has great potential for preventing the relapse of HER2+ breast tumors after breast-conserving surgery with enhanced therapeutic efficacy, improved patient compliance and significantly reduced side effects.

## Introduction

Breast cancer takes up a quarter of all cancers in females, and it has become the leading cause of cancer death globally among females 1-3. The treatment modalities of breast tumors include surgery, chemotherapy, radiotherapy, hormone-blocking therapy and immunotherapy, depending on the subtype, size and stage of the tumor. Thus far, surgical treatment remains the preferred treatment modality. Breast-conserving surgery, which is an operation to excise primary breast tumors and adjacent breast tissues while maintaining the shape and appearance of the breast 4 has been recognized as an ideal choice to replace mammectomy for appropriate candidates with early-stage mammary carcinoma 5. The advantages of breast-conserving therapy (BCT) compared to mammectomy include greatly diminished psychological burden, better cosmetic results, lower incidence of chest wall adhesions, and reduced wound infection risk 5, 6.

HER2 (ErbB2), which is a transmembrane receptor with tyrosine kinase activity 7, is amplified and/or overexpressed in one fifth to a quarter of mammary cancers 8. The HER2 overexpression has been shown to relevant to resistance to therapy, poorly differentiated high-grade tumors and an increased incidence of brain metastasis 8. In particular, patients with HER2+ breast tumors have a very poor prognosis after BCT, with a local relapse rate as high as 15.7%, which is several times greater than that of other subtypes of breast tumors, such as luminal subtype tumors and triple-negative tumors 9.

Immunotherapy, which harnesses the immune system to combat cancers, has developed rapidly in recent years 10-16. Herceptin is the first humanized monoclonal antibody licensed by the U.S. Food and Drug Administration (FDA) in 1998 for the targeted therapy of HER2+ breast cancer 8. Its global sale has increased to over $7 billion in 2017. Herceptin can direct against the extracellular domain of the HER2 protein, inhibit downstream signaling pathways, including PI3 kinase (PI3K) and MAP kinase (MAPK) cascades, activate antibody-dependent cellular cytotoxicity (ADCC) response, cause cell-cycle arrest and apoptosis, suppress angiogenesis and interfere with DNA repair 7, 8. The activation of ADCC response involves an increase in the infiltration of natural killer (NK) cells, a main immune cell type, and subsequent NK-cell mediated tumor cell lysis 17. Therefore, Herceptin can target HER2+ breast tumor cells and effectively prevent tumor relapse after BCT 18.

Intravenous administration is the main delivery mode for Herceptin in clinic, but this administration route suffers from some problems, such as cardiotoxicity, poor tumor retention and weekly administration 19-22. In 2013, the European Medicines Agency (EMA) approved the hypodermic injection of Herceptin for the treatment of HER2+ breast cancer 23. A previous phase III clinical trial demonstrated that the hypodermic administration of both Herceptin and recombinant human hyaluronidase (rHuPH20), which is an enzyme that degrades interstitial hyaluronic acid adjacent to the injection site, achieved a similar pharmacokinetic profile and therapeutic efficacy as the traditional intravenous administration route 24. The change in the delivery mode has facilitated great improvements in patient convenience and optimized the use of medical resources; however, the hypodermic administration route failed to overcome some problems, especially cardiotoxicity, that the intravenous administration of Herceptin faced.

Another valid treatment alternative that enhances the therapeutic efficacy while minimizing systemic side effects is the localized sustained delivery of drugs 25-30. In particular, injectable and thermosensitive hydrogels have been widely employed for localized drug delivery for the treatment of various diseases 31-35. In general, these types of materials are low-viscous sols at low or room temperature, which make it convenient to entrap fragile therapeutic agents, such as polypeptides 36, 37 and proteins 38, 39, by simply mixing them, and then transform into semisolid gels upon injection into the body due to the temperature-triggered sol-gel transition. Subsequently, loaded drugs can be released from the hydrogel depot in a predefined controlled pattern. Till now, block copolymers composed of PEG/polyester 40-45, PEG/polypeptide 46-49 and poly(phosphazenes) 50, 51 have been exploited as representatively biodegradable and temperature-responsive hydrogels. Among them, thermosensitive hydrogels composed of poly(lactic acid-*co*-glycolic acid)-*b*-poly(ethylene glycol)-*b*-poly(lactic acid-*co*-glycolic acid) (PLGA-PEG-PLGA) triblock copolymers gain in popularity because of convenient one-pot synthesis and a good safety profile 52-55. Additionally, both PLGA and PEG licensed by the FDA have been clinically utilized for many years.

Nonetheless, the composition window of PLGA-PEG-PLGA triblock copolymers to achieve a thermosensitive hydrogel is very narrow 56-58, causing them to fail to gratify the diversified requirements of biomedical applications. Once outside the suitable composition range, the polymers just form sols or precipitates in aqueous medium in the temperature window suitable for *in vivo* biomedical applications. In fact, a subtle equilibrium between hydrophilicity and hydrophobicity plays a crucial role in the temperature-induced sol-gel transition of these types of amphiphilic copolymers 57, 58. Inspired by this, a pragmatic blend approach has been exploited to construct thermosensitive hydrogels through blending an aqueous solution of a PLGA-PEG-PLGA copolymer with a precipitate, which is an analogue containing a different PEG/PLGA proportion 55, 59. This approach effectively broadens the available window of relevant polymers and opens a new avenue to design other thermosensitive hydrogels. Furthermore, both *in vitro* and *in vivo* experiments demonstrated that the PLGA-PEG-PLGA mixture thermosensitive hydrogels had good biocompatibility and tunable biodegradability 55.

**Figure 1:**
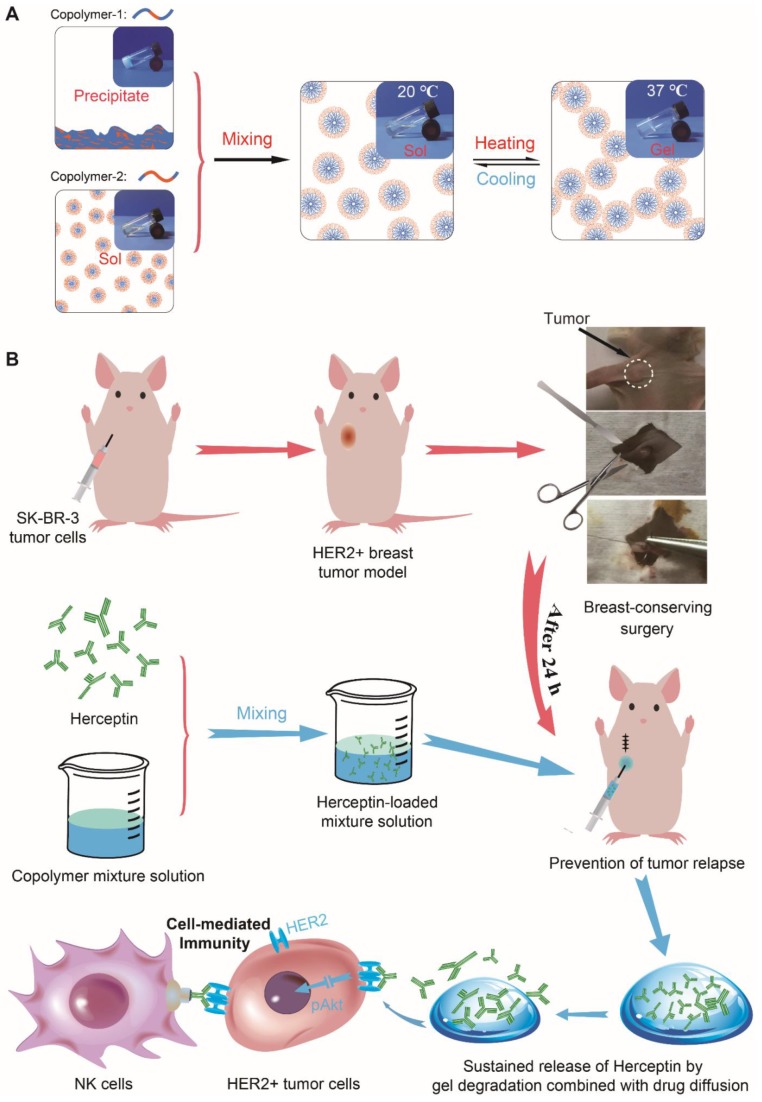
A Herceptin-loaded mixture hydrogel for the therapy of HER2+ breast tumors. A) Injectable and thermosensitive hydrogels formed by mixing a sol of a PLGA-PEG-PLGA triblock copolymer, which is actually a suspension of micelles, and a sediment of an analogue containing a different PEG/PLGA proportion. Their mixtures with rational mix proportions likewise form micelles in aqueous medium at low temperatures, and with an increase of temperature, the micellar aggregation driven by the hydrophobic interaction induces the formation of a percolated micelle network, the so-called sol-gel transition 59. B) A schematic of the Herceptin-loaded hydrogel for preventing the local relapse of HER2+ breast tumors after breast-conserving surgery. A HER2+ breast tumor model was first created in nude mice, and the tumors were then excised by imitative breast-conserving surgery. Approximately 1 mm^3^ of tumor mass was separated from the original tumor and then placed into the excision site to imitate the residual tumor after BCT. After one day of surgery, the Herceptin-loaded hydrogel was hypodermically injected, and the administration site was 5 mm away from the excision site of the tumor. The sustained release of Herceptin was achieved by gel degradation combined with drug diffusion. The pAkt signaling pathway was inhibited by the sustained delivery of Herceptin, and the NK cell-mediated immunity response was activated.

In the present work, we provided a novel strategy using thermosensitive hydrogels to achieve localized sustained delivery of Herceptin to reduce the risk of local relapse of HER2+ breast tumors after breast-conserving surgery while minimizing systemic side effects, especially cardiotoxicity. Injectable and thermosensitive PLGA-PEG-PLGA mixture hydrogels were constructed based on the blending approach. As shown in Figure 1A, copolymer-1 was precipitated in water, and copolymer-2 was dissolved in water; however, both copolymers failed to form a thermosensitive hydrogel as the temperature increased. Their mixtures with rational mix proportions displayed sol-gel transitions with rising of temperature. Herceptin was conveniently loaded by blending the antibody with the aqueous polymer solutions at a low temperature. Both *in vitro* and *in vivo* release profiles of Herceptin from the hydrogel depot were evaluated. The *in vitro* and *in vivo* degradation behaviours of the mixture hydrogels were also examined. The *in vivo* anticancer efficacy against SK-BR-3 tumor-bearing nude mice was detected by hypodermical injection of the Herceptin-loaded hydrogel system. Notably, some delivery systems of Herceptin have been exploited and have shown enhanced anticancer efficacy on HER2+ breast tumor-bearing mouse models 21, 22, 60; however, as far as we know, their potential cardiotoxicity has not been reported, and in clinical practice, Herceptin is mainly used to prevent local relapse after surgery. Therefore, the cardiotoxicity of the Herceptin-loaded hydrogel was analyzed by echocardiography for the first time, and to simulate clinical application, a relapse model of HER2+ breast tumors was also constructed by imitative breast-conserving surgery on nude mice and the anti-relapse efficacy was evaluated after administration of the abovementioned hydrogel formulation, as illustrated in Figure 1B.

## Results

### Synthesis and characterization of the PLGA-PEG-PLGA triblock copolymers

**Figure 2:**
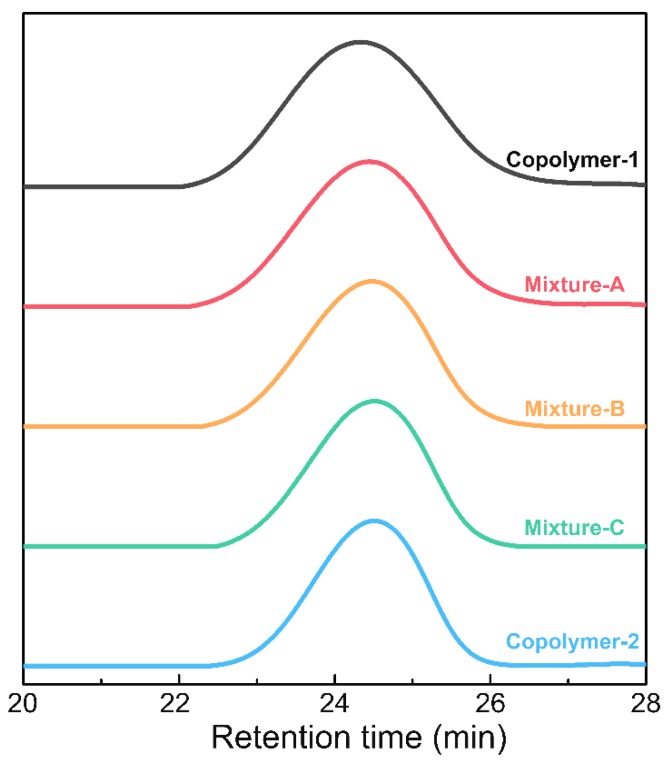
GPC traces of the two triblock copolymers and their indicated mixtures.

The synthesis of the PLGA-PEG-PLGA triblock copolymers was performed by bulk ring-opening copolymerization of D,L-lactide (LA) and glycolide (GA) using dihydroxy-terminal PEG as the macroinitiator with the help of the catalyst Sn(Oct)_2_. ^1^H nuclear magnetic resonance (NMR) and gel permeation chromatography (GPC) measurements were executed to determine the compositions and molecular weights (MWs) of the PLGA-PEG-PLGA triblock copolymers. The ^1^H NMR spectrum of copolymer-2 is presented in Figure S1. The characteristic peaks at 3.65, 4.80 and 5.25 ppm were integrated and then employed to calculate the number-averaged MWs and LA/GA molar proportions of the two PLGA-PEG-PLGA polymers 59. The results showed that the two samples exhibited the same molar proportion of LA/GA and total MWs but different PEG/PLGA block proportions. Furthermore, as shown in Figure 2, all the GPC traces of the two samples and their mixtures with various weight mix proportions presented a unimodal pattern, indicating that the two polymers with similar MWs were successfully synthesized. Table 1 summarizes the basic information about the two triblock copolymers and their indicated mixtures.

### Sol-gel transition of aqueous solutions of the copolymer mixtures

**Figure 3:**
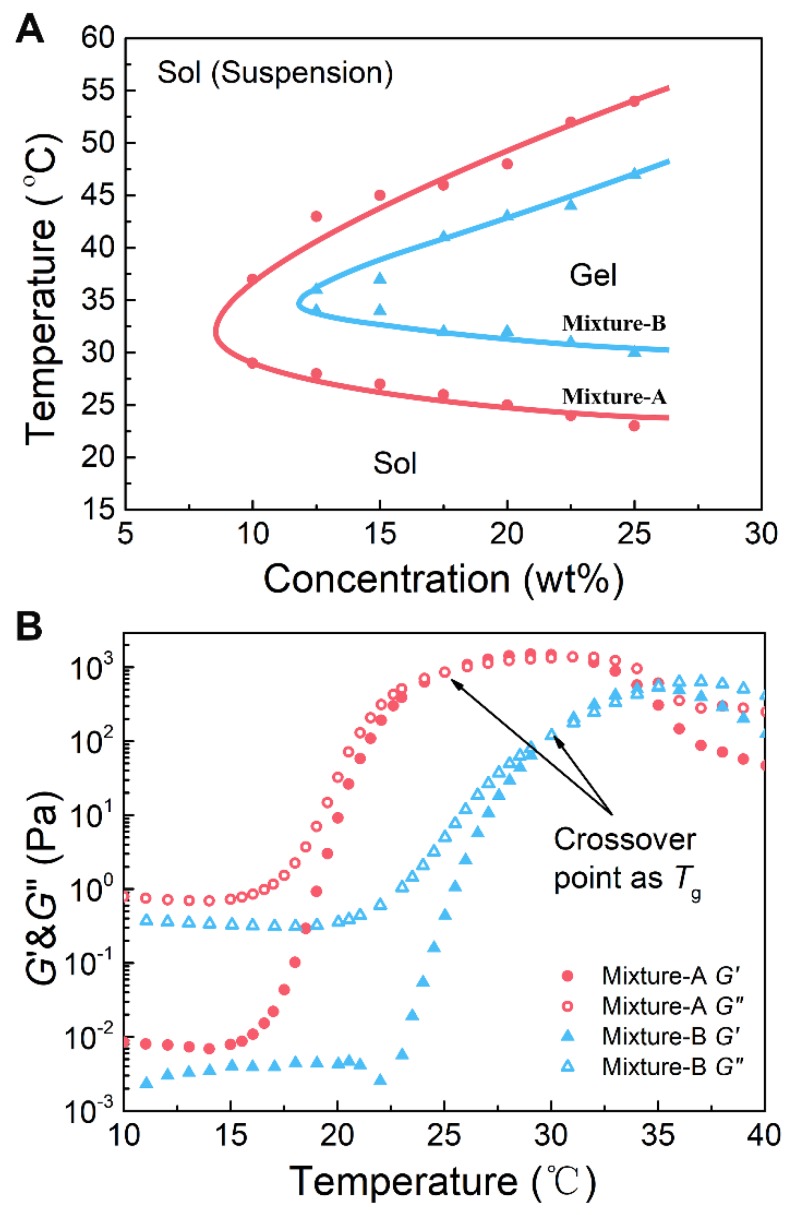
(A) Phase diagrams of the aqueous solutions of the copolymer mixtures with the indicated mix proportions. The transition temperature was determined by the vial-inverting approach. (B) The storage modulus (*G′*) and loss modulus (*G*′′) of the aqueous solutions of the copolymer mixtures as a function of temperature. Shear frequency: 1.59 Hz; heating rate: 0.5 ºC /min. The polymer mixture concentration was 25 wt%.

First, we tested the water solubility of the two polymers. As expected, copolymer-1 did not dissolve in water, whereas copolymer-2 was easily soluble in water. Neither copolymer formed a thermosensitive hydrogel in water; however, their mixtures with rational mix proportions were soluble in aqueous medium and exhibited sol-gel transitions with rising of temperature. This feature was attributed to a subtle total equilibrium of hydrophilicity and hydrophobicity of those mixtures. The sol-gel transition temperatures (*T*_g_s) of the aqueous solutions of the copolymer mixtures with various mix proportions and polymer concentrations were confirmed by the vial-inverting approach. The aqueous solutions of mixture-C at all polymer concentrations failed to form an* in situ* hydrogel upon heating because the samples contained too high of a content of the hydrophilic copolymer-2. The aqueous solutions of mixture-A and mixture-B showed three different physical states (sol, gel, and sol (suspension)) with increasing temperature from 20 to 60 ºC, as shown in Figure 3A. The mixture-A/water system exhibited a broader gel window and a lower critical gel concentration than the mixture-B/water system. Importantly, the gel windows of the two mixture/water systems covered body temperature, and the *T*_g_ could be easily tailored from 23 to 31 ºC, which is a physiologically important temperature window, by simply adjusting the mix proportion from 7:3 to 5:5. These results indicated that the mixture hydrogel systems with an easily adjustable *T*_g_ are able to meet different requirements of biomedical applications.

Changes in the storage modulus (*G*′) and loss modulus (*G*′′) of the aqueous solutions (25 wt%) of the copolymer mixtures with an increase in temperature were monitored by dynamic rheological analysis, and the results are shown in Figure 3B. At low temperatures, the *G*′ and *G′*′ of the polymer solutions were low, and the *G*′′ was obviously greater than the *G*′, suggesting a typical liquid state. Sharp increases in both* G*′ and *G*′′ were observed at a certain temperature, and the level of the increase in *G*′ was greater than the level of the increase in *G*′′, which indicated the occurrence of* in situ* gelation. Generally, when the *G*′ is equal to the *G*′′ in rheology, the corresponding temperature is defined as *T*_g_ 54. The *T*_g_s, which were very close to those confirmed by the vial-inverting approach, were found to be 24 and 31 °C for mixture-A and mixture-B, respectively. In addition, by changing the mix proportion from 7:3 to 5:5, the *T*_g_ increased; however, the maximum values of *G*′ and *G*′′ decreased. As presented in Figure S2B, for the aqueous solution of mixture-C, no crossover point between the *G*′ and *G*′′ was detected with increasing temperature. Namely, mixture-C was not able to form a physical hydrogel at any temperature, which is also consistent with the result obtained from the phase diagram measurement. Consequently, the mixture-A and mixture-B hydrogels were employed in the subsequent studies.

### *In vitro* release of Herceptin

**Figure 4:**
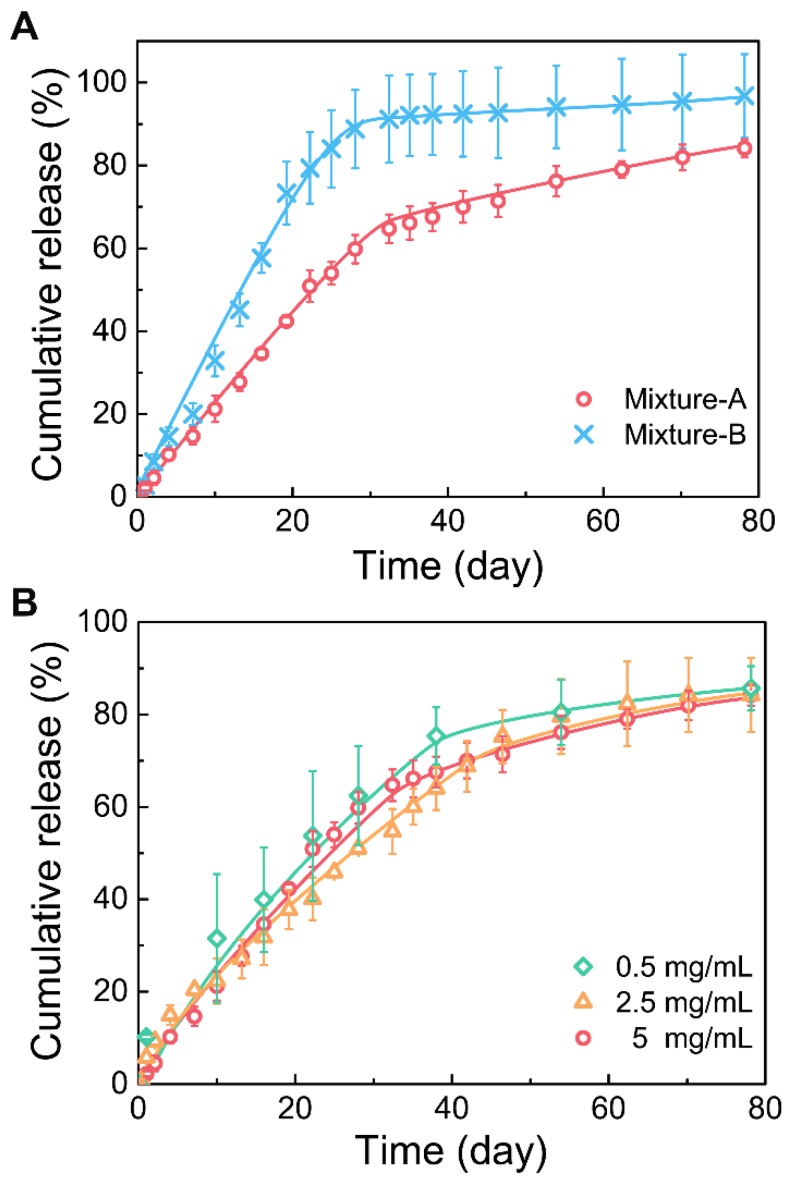
(A) Release curves of Herceptin from the hydrogel systems with different mix proportions in phosphate-buffered saline (PBS) at 37 ºC. The concentration of polymer mixture was 25 wt%, and the drug concentration was 5 mg/mL. (B) Release curves of Herceptin from the mixture-A hydrogel systems with different drug concentrations in PBS at 37 °C. The concentration of polymer mixture was 25 wt%.

First, the *in vitro* Herceptin release profiles from different mixture hydrogels were evaluated (Figure 4A). Both the hydrogel formulations showed a two-phase drug release manner. At the first stage, Herceptin was continuously released at a nearly constant rate followed by a relatively slow release profile in the second stage. The effective release of Herceptin from the mixture-B hydrogel was detected only in the first stage, and the total amount of the drug released was more than 90% within this stage, suggesting that the release of Herceptin in the second stage was nearly negligible. Due to the mixture-A system containing more of the hydrophobic component copolymer-1, the release rate of Herceptin from the mixture-A hydrogel was slower than the mixture-B hydrogel at the first stage, but the Herceptin-loaded mixture-A hydrogel exhibited a higher release rate at the second stage than the mixture-B hydrogel formulation. As a result, the sustained release of Herceptin from the mixture-A hydrogel persisted for over 80 days, as far as we know, which is the longest period of Herceptin delivery from a delivery system compared to those have ever been reported 21, 22, 60, 61.

The influence of drug loading amounts on the drug release kinetics was further studied and the results are shown in Figure 4B. No significant effects on the drug release curves of the mixture-A hydrogel systems were observed with changing the drug loading amount from 0.5 mg/mL to 5 mg/mL. This finding manifested that the mixture-A hydrogel is very appropriate for the sustained delivery of Herceptin, and the required drug loading amount is facilely chosen without affecting the drug release profile.

**Figure 5:**
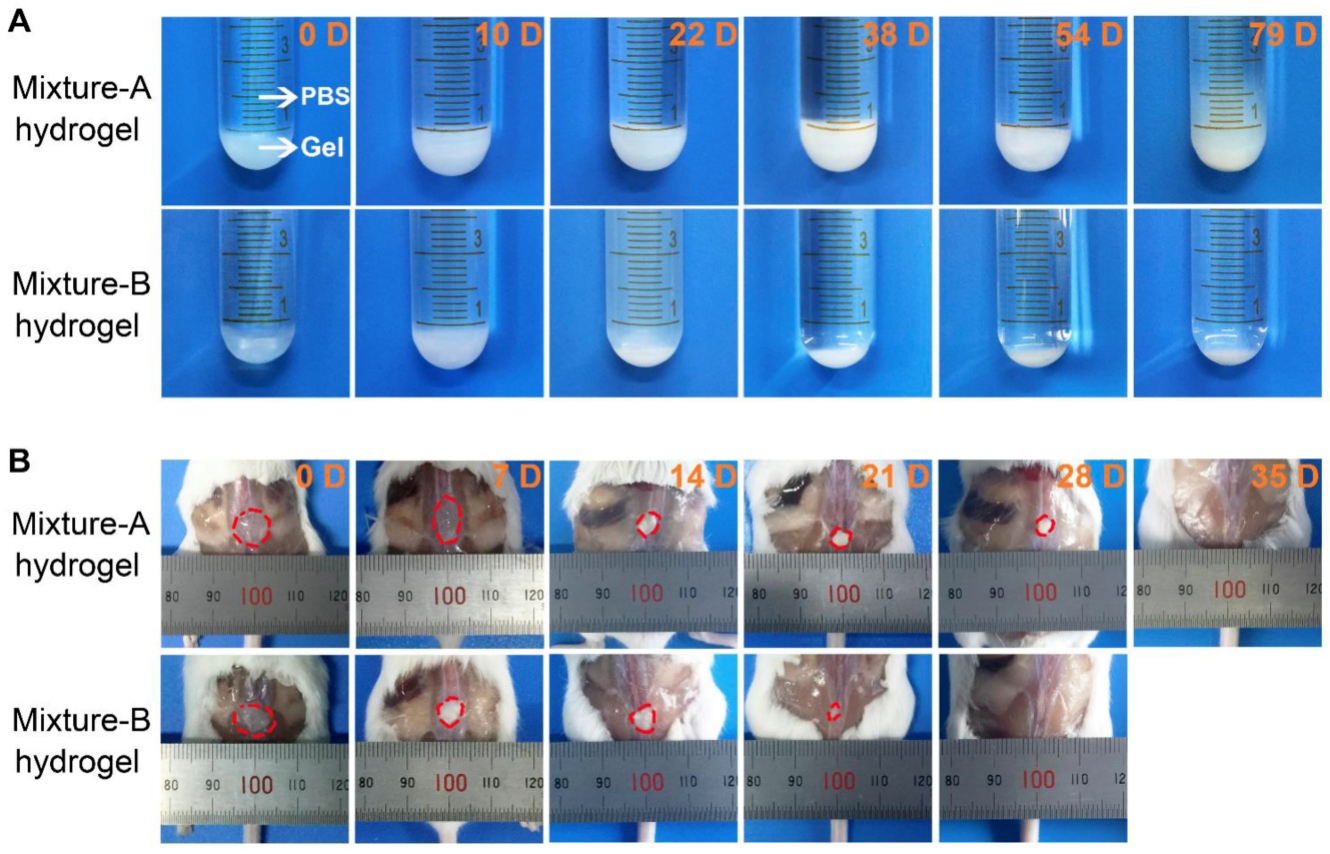
(A) Optical images of the Herceptin-loaded mixture hydrogels at the fixed degradation time points in PBS at 37 ℃. (B) Optical images of the mixture hydrogels at the fixed degradation time points *in vivo*. The initial polymer concentration was 25 wt%.

The morphological changes of the mixture-A and mixture-B hydrogels over the time of drug release were also observed during the *in vitro* release tests. As displayed in Figure 5A, the integrity of the opaque mixture-A hydrogel containing Herceptin was maintained for up to 54 days, followed by the collapse of the gel skeleton. In contrast, the Herceptin-loaded mixture-B hydrogel exhibited a translucent state at the initial stage and then changed into an opaque state following the degradation of the hydrogel matrix. The integrity of the Herceptin-loaded mixture-B hydrogel lasted only 2-3 weeks. The differences in the *in vitro* degradation behaviours of the two hydrogels were attributed to their different mix proportions. The mixture-A hydrogel, which contained more of the hydrophobic component copolymer-1, had a slower degradation rate than the mixture-B hydrogel. This change trend was consistent with the release profiles of Herceptin *in vitro*, indicating that the gel degradation combined with drug diffusion governed the release of Herceptin.

### *In vivo* degradation of the mixture hydrogels

As an implanted biomaterial, its *in vivo* degradation should be concerned and investigated. Aqueous 25 wt% solutions of mixture-A and mixture-B were injected into the subcutaneous layers of the dorsal areas of ICR mice using a conventional syringe, and the hydrogels rapidly formed *in situ* at the administration sites because of contacting with the body heat. As shown in Figure 5B, the *in vivo* persistence of the mixture-A hydrogel lasted 4-5 weeks, while the *in vivo* maintenance of the mixture-B hydrogel decreased at approximately 3 weeks, and only a bit residual hydrogel was seen on day 21. This varying trend coincided with the *in vitro* change in the hydrogel morphology. Nevertheless, the *in vivo* degradation rate was obviously faster than that *in vitro* due to the existence of lipase and a complicated biological environment, which significantly accelerated the *in vivo* degradation of the hydrogel.

### *In vivo* behaviours of Herceptin-loaded hydrogels

**Figure 6:**
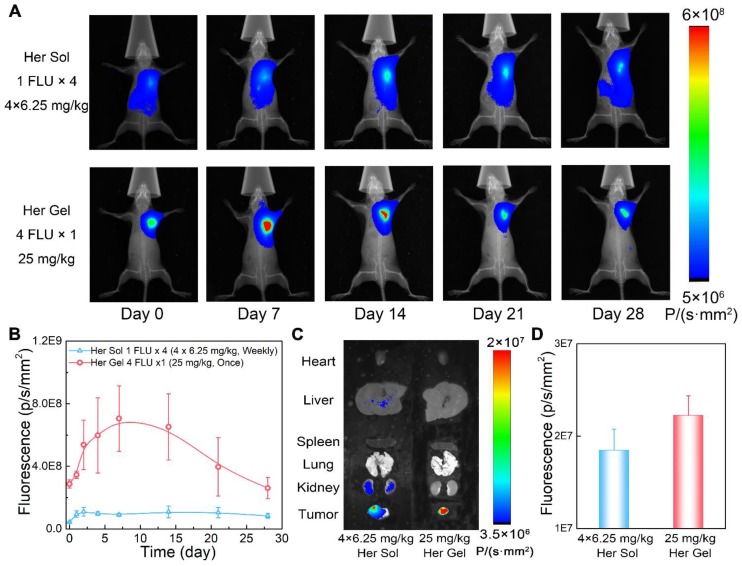
*In vivo* behaviours of the Herceptin-loaded mixture-A hydrogel. (A) Real-time *in vivo* fluorescent imaging of SK-BR-3 tumor-bearing nude mice with hypodermic injection of Cy5.5-Her solution or the Cy5.5-Her-loaded mixture-A hydrogel. The fluorescence intensity of Cy5.5-Her in the hydrogel was 4 times greater than that of Cy5.5-Her solution; however, the Cy5.5-Her solution was injected into the mice once per week for 4 weeks, and the Cy5.5-Her-loaded hydrogel was injected only once. Therefore, the total amount of Cy5.5-Her in the two groups was equal. All the images shown in the same group are from the same mouse. (B) Semiquantitative analysis of the fluorescence signals at the injection sites as a function of time. The first injection time was defined as day 0. (C) *Ex vivo* fluorescent imaging of the major organs and tumors harvested from the mice on day 28 post-treatment. (D) Semiquantitative analysis of the fluorescence signals in tumors on day 28 post-treatment.

Combined with the *in vitro* release profiles and the *in vivo* degradation data, the Herceptin-loaded mixture-A hydrogel was chosen as the optimal delivery system for the* in vivo* studies. First, we evaluated the *in vivo* Herceptin release behaviours from the hydrogel matrix by labeling the antibody with Cyanine5.5 (Cy5.5), which is a commonly used fluorescent imaging probe. The aqueous solution of mixture-A containing Cy5.5-labeled Herceptin (Cy5.5-Her) was hypodermically injected into SK-BR-3 tumor-bearing nude mice. The injection site was 5 mm away from the tumor, and Herceptin solution, which was hypodermically injected once per week for four weeks, was set as a control. Under *in vivo* fluorescent imaging, the injection site maintained strong fluorescence signals during the observation period of 4 weeks after a single injection of the hydrogel system, as displayed in Figure 6A. In contrast, relatively weak fluorescence signals were observed at the injection site after weekly injections of the Cy5.5-Her solution.

Figure 6B shows the semiquantitative data of the fluorescence intensity as a function of time. The fluorescence intensity first enhanced and then decreased in the mice injected with the Cy5.5-Her-loaded hydrogel over time, reflecting the slow and constant release of the drug out of the hydrogel depot. The fluorescence intensity was positively correlated with the tissue depth of the fluorescence molecule and the enhancement of the fluorescence intensity at the initial stage was attributed to the diffusion of fluorescence molecule to the surrounding skin tissue. The fluorescence intensity in the Cy5.5-Her solution group was obviously weaker than that in the Cy5.5-Her-loaded hydrogel group. This feature was ascribed to the rapid diffusion of the Cy5.5-Her solution after injection under hypodermic tissue. At 4 weeks post-treatment, the major organs and tumor tissues were collected for *ex vivo* fluorescent imaging to detect and compare the biodistribution of Cy5.5-Her in different tissues. Previous studies have revealed that intravenous administration of Herceptin leads to the accumulation of the drug mainly within organs such as kidneys, livers and lungs 21. In contrast, as displayed in Figure 6C and 6D, the fluorescence intensity in the tumors of mice treated with Cy5.5-Her solution or the Cy5.5-Her-loaded hydrogel was significantly higher than in the major organs of the mice. This finding was most likely attributed to the proximity of the injection site to the tumor tissue, which might allow for accessible diffusion of the antibody into the tumor. Furthermore, compared to the Cy5.5-Her solution group, the Cy5.5-Her-loaded hydrogel group exhibited stronger fluorescence signals in the tumors but weaker fluorescence signals in the major organs, indicating a higher accumulation of the antibody in the tumors of the mice treated with the hydrogel formulation.

Furthermore, both the Herceptin-loaded hydrogel and Herceptin solution containing the same amount of Cy5.5-Her were tested *in vivo* for a week after a single injection of Cy5.5-Her-loaded hydrogel or Cy5.5-Her solution, and the results are displayed in Figure S3. The fluorescence intensity of the Cy5.5-Her-loaded hydrogel exhibited a steady increase during the one-week examination period. In contrast to the hydrogel formulation, the fluorescence intensity in the Cy5.5-Her solution group first increased and then fell within seven days, which coincided with the *in vivo* half-life of Herceptin (5.3 days) 60.

### *In vivo* anticancer efficacy

**Figure 7:**
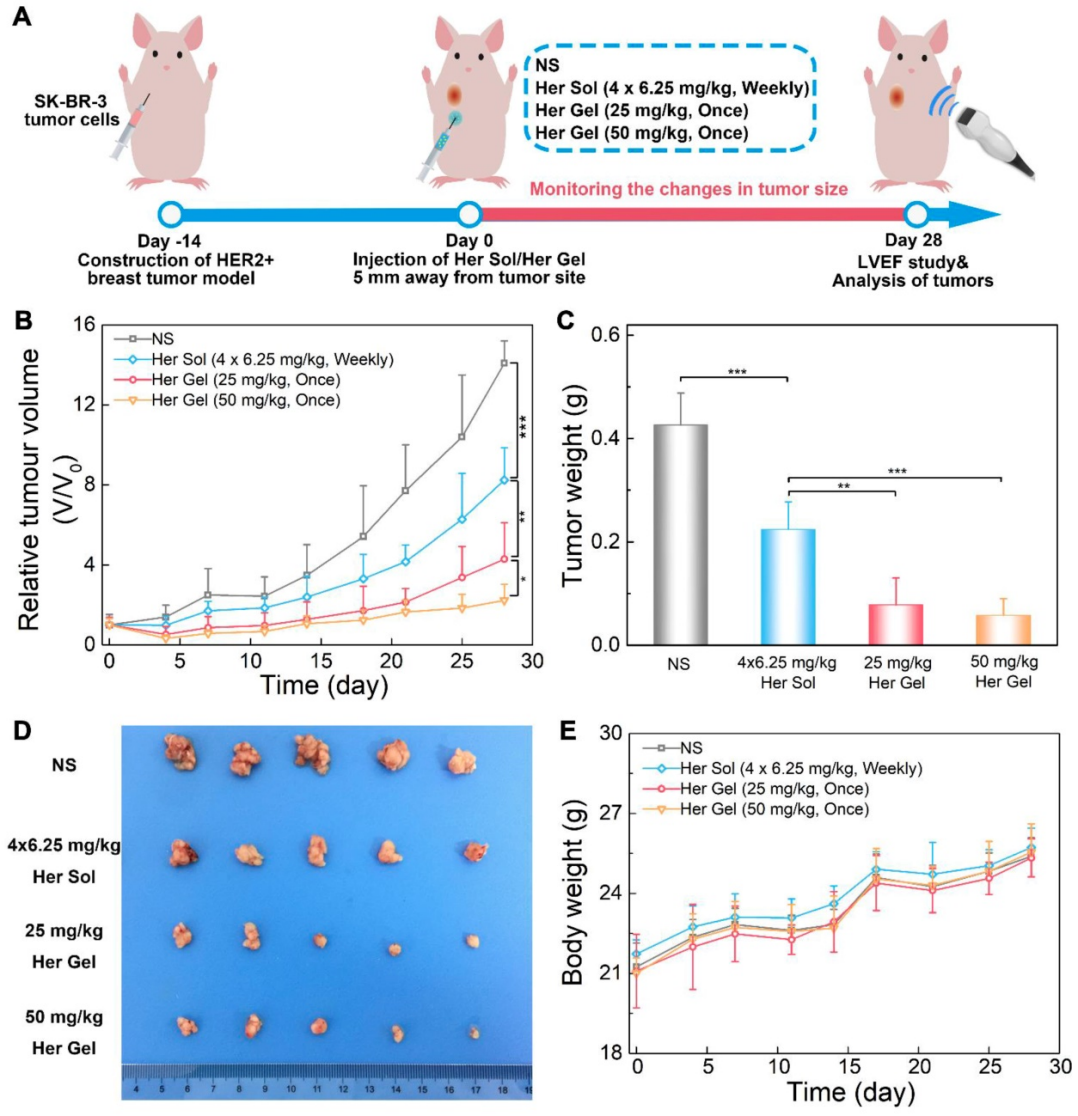
*In vivo* anticancer efficacy. (A) A schematic showing the experimental procedure for investigating the *in vivo* anticancer effect in a HER2+ breast tumor mouse model. (B) Changes in tumor volume of mice receiving the various treatments over time. *V* and *V*_0_ indicate the tumor volumes after and before the treatment, respectively. (C)* Ex vivo* tumor weight on day 28 post-treatment. (D) Tumors imaged by a digital camera. (E) Body weights of mice after various treatments. Each point represents the mean ±SD; *n* =5.

The *in vivo* anticancer efficacy of the Herceptin-loaded mixture-A hydrogel formulation was evaluated in SK-BR-3 tumor-bearing nude mice, and Figure 7A schematically shows the experimental procedure. As presented in Figure 7B, the tumor volume increased rapidly over time in the control (normal saline, NS) group. Both the Herceptin solution and Herceptin-loaded hydrogels effectively inhibited tumor growth. Compared to the treatment by weekly injections of Herceptin solution, a single administration of the Herceptin-loaded hydrogel at the same dosage of the drug seemed to be more efficient to suppress tumor growth. Meanwhile, the effect on the inhibition of tumor growth was dependent on the dose of the antibody. A single administration of the hydrogel system containing 50 mg/kg Herceptin exhibited the most effective inhibition of tumor growth.

28 days later, all the mice were euthanized, and the weights of tumors harvested were recorded. The final average weights of the tumors from the mice that received different treatments were in good agreement with the changes in the tumor volume, as shown in Figure 7C-D. A single injection of 25 mg/kg Herceptin-loaded hydrogel exhibited a better antitumor effect than weekly injections of 6.25 mg/kg Herceptin solution within 4 weeks. Although the administration of the hydrogel system containing 50 mg/kg Herceptin resulted in the smallest average weight of the tumor, no significant difference was observed between the two groups of the Herceptin-loaded hydrogels with various doses of the antibody.

The body weights of the nude mice were also monitored as a function of time as a signal of the systematic toxicity of the various treatments. All the animals that received different treatments exhibited continuous increases in the body weights (Figure 7E), indicating that the different treatments did not appear to cause significant systematic toxicity during the test period.

The sustained utilization of Herceptin may result in a gradual decay in the left ventricular ejection fraction (LVEF) and heart failure, which is one of the crucial concerns that limit the clinical application of Herceptin 19, 20. Currently, echocardiography is utilized to examine the decrease in the LVEF in clinic 62-64. Whether Herceptin therapy should be continued or discontinued depends on the reduction extent of LVEF 64.

**Figure 8:**
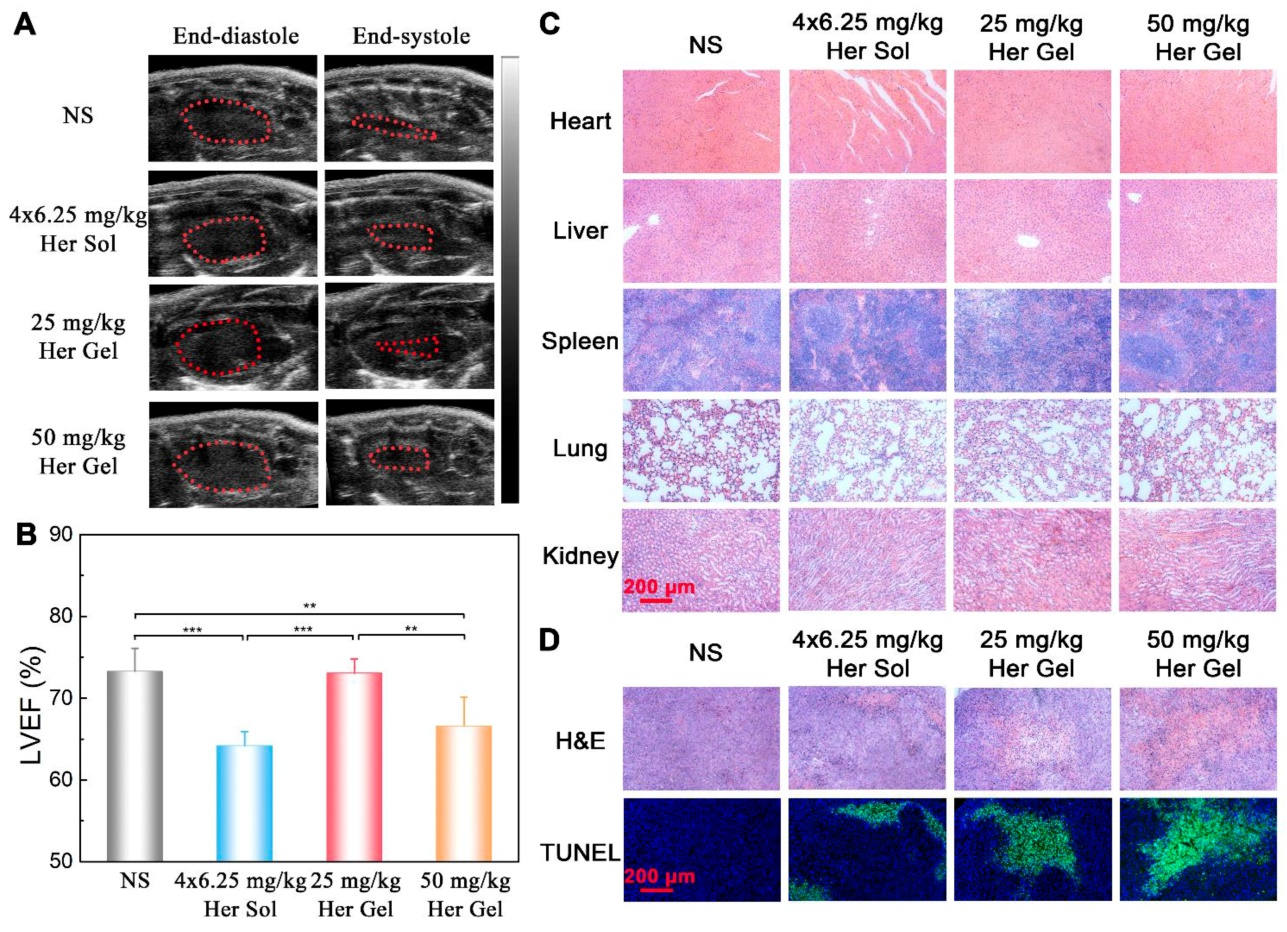
(A) Representative left ventricular end-diastole images and end-systole images of mice after various treatments. The dashed lines represent the left ventricle area. (B) Average LVEF values of nude mice in the different groups. (C) Representative micrographs of H&E-stained slices of major organs of nude mice in the different groups. (D) Representative micrographs of H&E- and TUNEL-stained slices of tumors harvested from nude mice in different groups. The region of TUNEL staining was the same as that of H&E staining.

Before sacrificing the animals (on day 28 post-treatment), the cardiac functions of the mice were measured by echocardiography. As shown in Figure 8A, the ratio of the left ventricular end-systolic volume to the left ventricular end-diastolic volume in both the Herceptin solution group and the 50 mg/kg Herceptin-loaded hydrogel group was obviously greater than those of the control group and 25 mg/kg Herceptin-loaded hydrogel group, indicating that the multiple injections of 6.25 mg/kg Herceptin solution and the single administration of 50 mg/kg Herceptin-loaded hydrogel caused cardiotoxicity to some extent. To further confirm this outcome, the LVEF values of the different groups were calculated. As shown in Figure 8B, the LVEF values in the Herceptin solution group and the 50 mg/kg Herceptin-loaded hydrogel group were significantly less than that in the control group. In contrast, the LVEF value in the 25 mg/kg Herceptin-loaded hydrogel group did not exhibit any difference from the control group, indicating that this treatment did not compromise cardiac function.

Subsequently, all the mice were euthanized, and their major organs and tumor tissues were dissected and collected. The autopsy showed no occurrence of tumor metastasis. This finding was attributed to the tumor model itself and the short modeling time. The specimens were sectioned and stained with hematoxylin/eosin (H&E) for histological analysis. No obvious abnormalities were detected in all of organs, as shown in Figure 8C. H&E staining and terminal deoxy-nucleotidyl transferase-mediated dUTP-biotin nick end labeling (TUNEL) assays of the tumor tissues were also performed to evaluate the degree of the apoptosis of tumor cells 7, 8. Different degrees of tumor necrosis, such as karyolysis, pyknosis, and karyorrhexis, were detected in the tumor tissues receiving the treatment of Herceptin solution or the hydrogel formulations (Figure 8D). Apparently, treatment with the 25 mg/kg Herceptin-loaded hydrogel led to higher tumor necrosis than treatment with the 6.25 mg/kg Herceptin solution. The TUNEL assays further revealed that there were higher apoptosis proportions of tumor cells in the Herceptin-loaded hydrogel groups compared to the Herceptin solution group.

**Figure 9:**
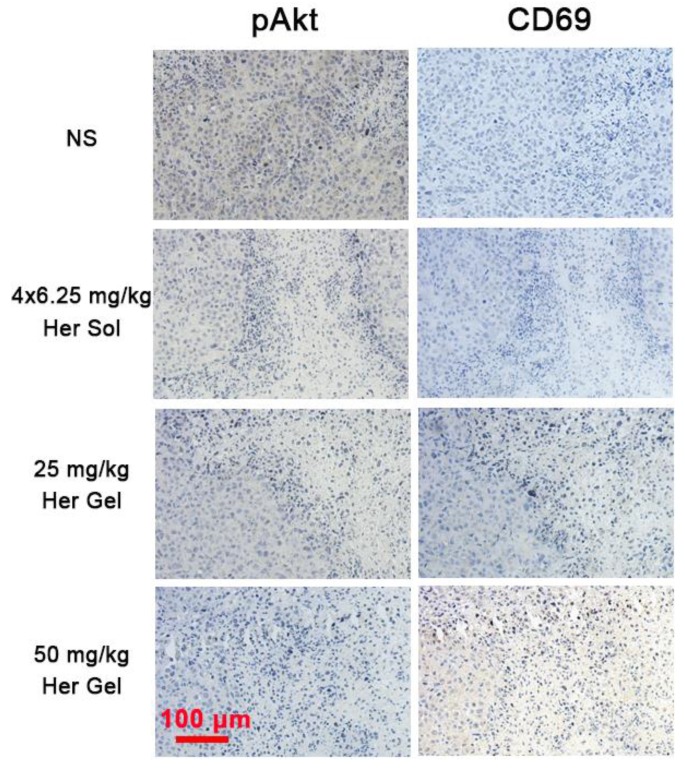
Representative micrographs of pAkt- and CD69-stained slices of tumors harvested from the nude mice in the different groups. The region of pAkt staining was the same as that of CD69 staining.

To further understand the immune action mechanism of Herceptin against HER2+ breast tumors, immunohistochemical staining of pAkt and CD69 was performed on the tumor tissues. The antitumor function of Herceptin includes activating the PI3K inhibitor PTEN, which leads to rapid Akt dephosphorylation and inhibition of cell proliferation, and inducing an ADCC response, which involves an increment in the infiltration of NK cells and subsequent NK cell-mediated tumor cell lysis 17. pAKT is phosphorylated Akt, and CD69 is a functional triggering molecule that activates NK cells 65. The brown color of 3,3'-diaminobenzidine (DAB) indicates pAkt-positive/CD69-positive signals. As shown in Figure 9, the strong brown DAB staining reflected the high expression level of pAkt in the control group, indicating that the proliferation of HER2+ tumor cells was active. In contrast, different degrees of inhibition of pAkt were observed in the Herceptin-treated groups. The CD69 staining results were the opposite of the feature of pAkt staining. The DAB staining intensity in the control group was very weak, implying the absence of infiltrated NK cells. Unlike the control group, the DAB staining intensities in the mice treated with the Herceptin-loaded hydrogels were markedly enhanced, suggesting an increase in infiltrated NK cells. Apparently, the mice receiving the 50 mg/kg Herceptin-loaded hydrogel treatment exhibited the strongest Akt dephosphorylation and highest ADCC activity, which well coincided with the inhibition efficacy on tumor growth, as shown in Figure 7B-C.

### *In vivo* anti-relapse efficacy after imitative breast-conserving surgery

**Figure 10:**
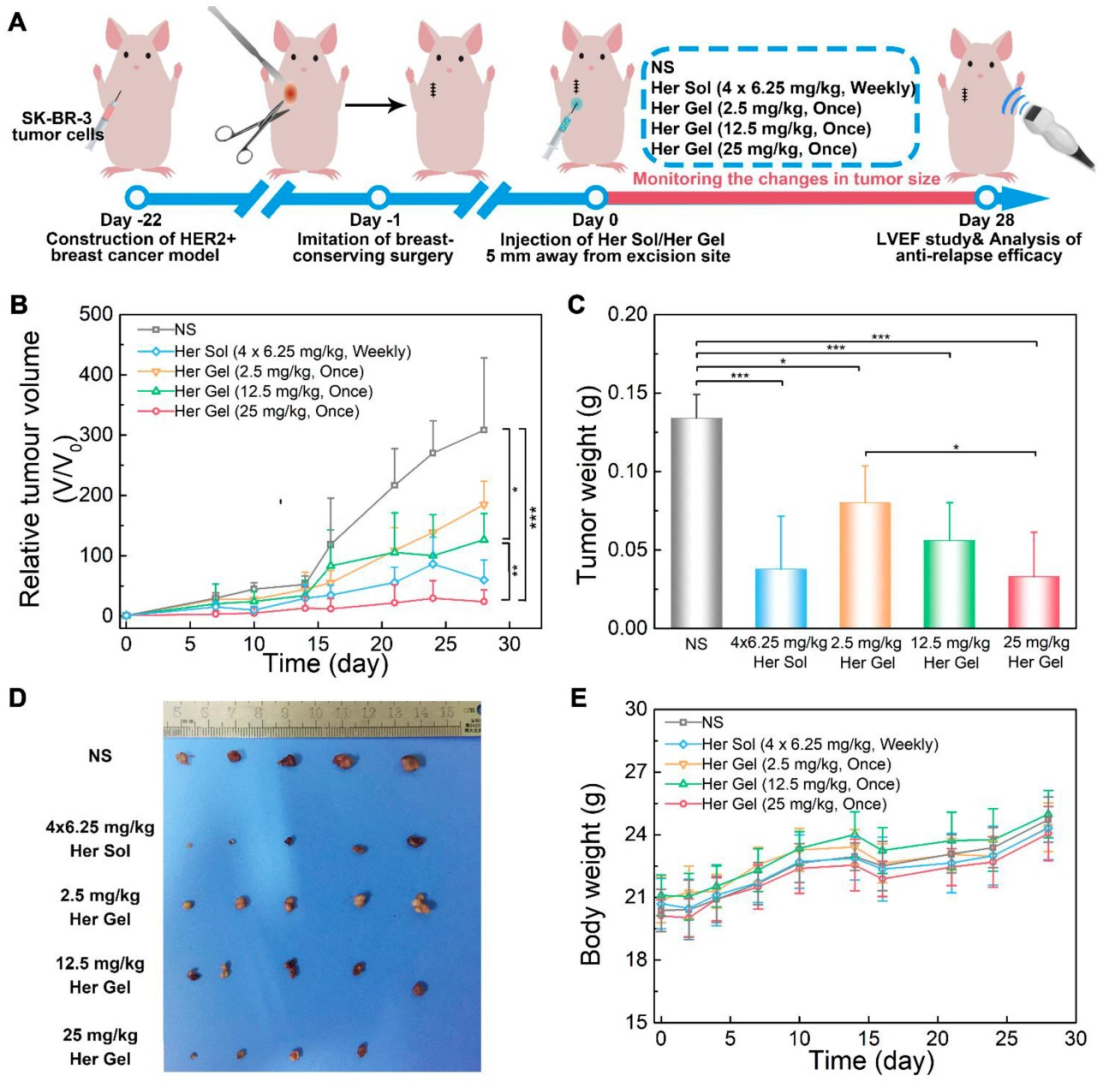
*In vivo* anti-relapse efficacy after imitative breast-conserving surgery. (A) A schematic showing the establishment of a HER2+ breast tumor relapse model in nude mice and the subsequent* in vivo* experimental procedure for investigating anti-relapse. (B) Changes in tumor volume of mice receiving the indicated treatments over time. *V* and *V*_0_ indicate the tumor volumes after and before the treatment, respectively. (C)* Ex vivo* tumor weight on day 28 post-treatment. (D) Tumors imaged by a digital camera. (E) Body weights of mice after the various treatments. Each point represents the mean ±SD; *n* = 5.

In fact, Herceptin is mainly utilized to prevent local relapse of HER2+ breast tumors after surgery in clinic 66. Therefore, to simulate the use of Herceptin in clinical practice, a SK-BR-3 tumor-bearing nude mouse model was first constructed, and an imitative breast-conserving surgery was then performed after the primary tumor volume reached ~100 mm^3^, followed by replanting a small amount of residual tumor. The nude mice received different treatments one day after surgery, and the injection site of the Herceptin-loaded hydrogel was 5 mm away from the excision site of the tumor, which was to facilitate observation of recurrent tumor, as illustrated in Figure 10A.

In the control group, the tumor volume run a very slowly rising tendency in the initial two weeks (Figure 10B). However, in the following two weeks, a tumor growth spurt was observed, and the final volume of the tumor reached approximately 300 times of the initial volume of the tumor. This feature indicated that the residual tumor resulted in the local relapse of the tumor. The administration of the Herceptin-loaded mixture-A hydrogels and the subsequent sustained release of Herceptin were able to inhibit tumor relapse, and the anti-relapse efficacy was markedly enhanced by increasing the Herceptin dosage. The exciting thing was that a single injection of the 25 mg/kg Herceptin-loaded hydrogel thoroughly suppressed the tumor relapse in the whole test period, and even the tumor of one nude mouse fully disappeared. The weekly injection of 6.25 mg/kg Herceptin solution also showed excellent anti-relapse efficacy. Notably, the postoperative complications, such as edema, made it difficult to measure the length and width of the tumor; therefore, the volume of the tumor in the first week after surgery was not recorded. These postoperative complications are also common after surgery in humans 67.

As shown in Figure 10C-D, the average tumor weight obtained 4 weeks post-treatment further demonstrated that both the Herceptin solution and Herceptin-loaded hydrogel at the same dosage of the antibody (25 mg/kg) successfully suppressed tumor relapse. Furthermore, all the body weights of the mice receiving Herceptin treatment showed the same increasing trend as the control group (Figure 10E).

**Figure 11:**
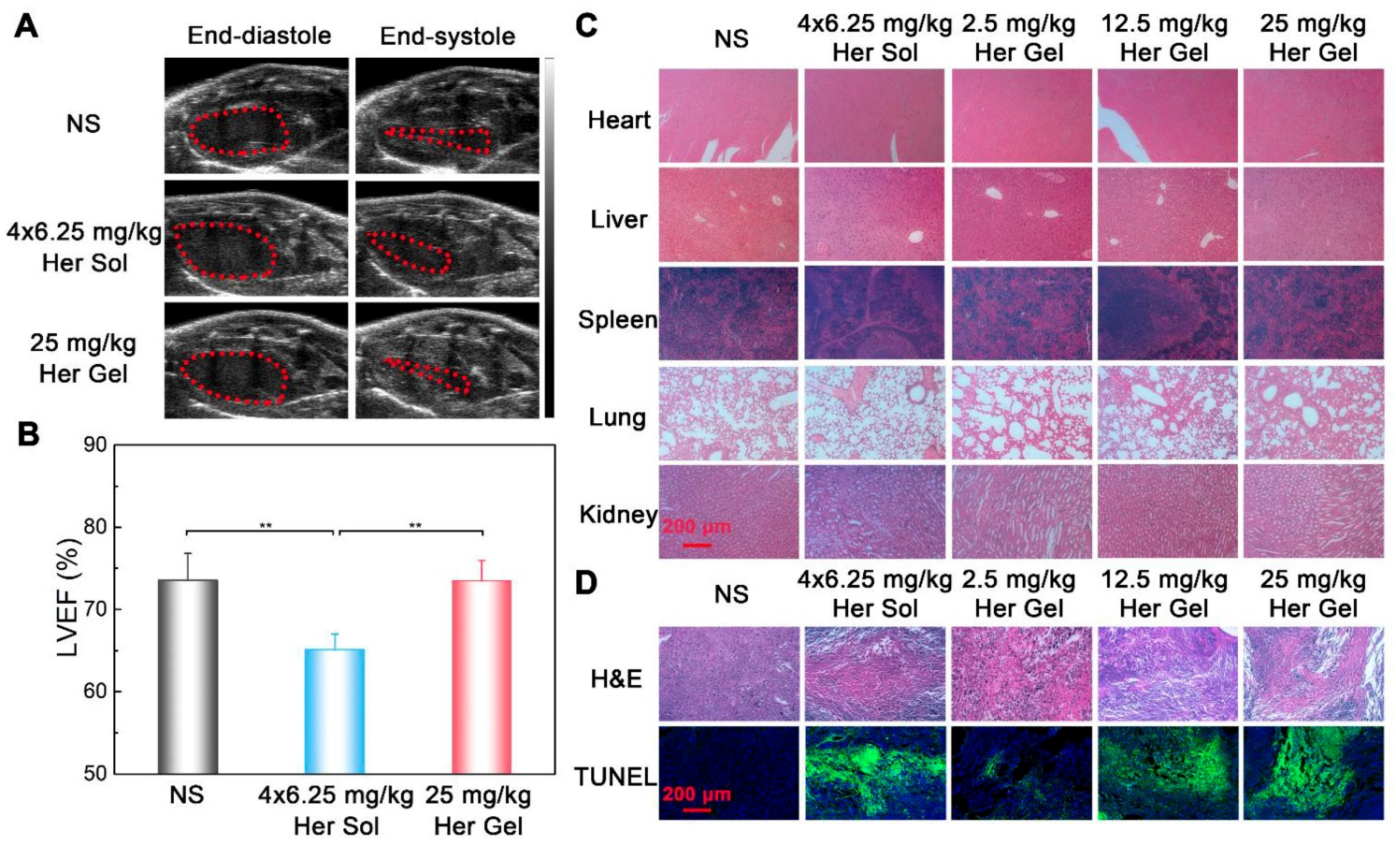
(A) Representative left ventricular end-diastole images and end-systole images of mice after the various treatments. The dashed lines represent the left ventricle area. (B) Average LVEF values of nude mice in the different groups. (C) Representative micrographs of H&E-stained slices of major organs of nude mice from the different groups. (D) Representative micrographs of H&E- and TUNEL-stained slices of tumors obtained from nude mice in the different groups. The region of TUNEL staining was the same as the region of H&E staining.

Before euthanasia of the animals, the cardiac functions of the mice were also measured. Consistent with the *in vivo* anticancer efficacy results, compared to the control group, the weekly injection of the Herceptin solution caused a significant decrease in the LVEF value, as shown in Figure 11A-B. In contrast, the sustained delivery of Herceptin from the hydrogel matrix did not cause an obvious change in the LVEF value, indicating that the mice had normal cardiac functions.

Finally, all the mice were euthanized, and their major organs and tumor tissues were dissected and collected. As presented in Figure 11C, all of the major organs showed a normal histological morphology, which was consistent with the experimental results from the *in vivo* antitumor efficacy analysis. Based on H&E staining, different levels of morphological changes and necrosis of the tumor tissues were detected after the mice received the treatment of the hydrogels containing various dosages of Herceptin (Figure 11D). We found the most substantial morphological changes and necrosis of the tumor slices obtained from the mice treated with the 25 mg/kg Herceptin-loaded hydrogel and the Herceptin solution. The TUNEL assays further revealed that the number of apoptotic tumor cells was proportional to the loading dosage of Herceptin in the hydrogel matrix, and the 25 mg/kg Herceptin-loaded hydrogel group and the Herceptin solution group induced the most substantial apoptosis of tumor cells.

## Discussion

To date, surgery remains the first choice for most patients in the treatment of breast cancers 66. Compared to a mammectomy, BCT has become increasingly popular for patients with early-stage mammary carcinoma due to the lower psychological burden and better cosmetic results 5, 6. Nevertheless, compared to other subtypes of breast tumors, such as luminal subtype tumors and triple-negative tumors, HER2+ breast tumors have a higher risk of local relapse following BCT 9. In clinic, weekly intravenous injections of Herceptin solution have been extensively utilized to avoid local relapse of HER2+ breast tumors after BCT, and the typically recommended duration of treatment is one year 68. However, cardiotoxicity, poor drug retention in tumors and frequent administrations are the main challenges associated with the intravenous injection of Herceptin 19-22. Consequently, it is important and relevant to develop a new delivery system of Herceptin with a higher therapeutic efficacy, less cardiotoxicity and better patient compliance.

In the present work, we developed a new strategy using injectable hydrogels to achieve localized long-term release of Herceptin for preventing local relapse of HER2+ breast tumors after BCT while minimizing cardiotoxicity. Injectable and thermosensitive PLGA-PEG-PLGA hydrogels with an adjustable gel performance and degradation rate were constructed by facilely mixing two PLGA-PEG-PLGA triblock copolymers with similar MWs but with different PEG/PLGA molar proportions, and the mixture-A hydrogel with a mix proportion of 7:3 and the mixture-B hydrogel with a mix proportion of 5:5 were then tried to deliver Herceptin.

Compared to the mixture-B hydrogel, the mixture-A hydrogel, which contained more of the hydrophobic component copolymer-1, exhibited a lower *T*_g_ (Figure 3A), a higher gel strength (Figure 3B) and a longer gel persistence *in vitro* and *in vivo* (Figure 5). These differences between the two hydrogel systems were attributed to their different global hydrophilic/hydrophobic proportions, which further influenced the release kinetics of Herceptin (Figure 4A). In particular, in the absence of an initial burst release, the Herceptin-loaded mixture-A hydrogel system achieved the sustained release of Herceptin *in vitro* for up to 80 days, which is the longest period of Herceptin delivery that has ever been reported. Furthermore, the mixture-A hydrogels with different drug loading amounts exhibited similar *in vitro* release profiles. This outcome manifested that the degradation of polymeric carrier rather than the drug loading amount governed Herceptin release. All the results indicated that the mixture-A hydrogel system is a better vehicle for the delivery of Herceptin than the mixture-B hydrogel system.

To noninvasively monitor the *in vivo* release of Herceptin from the hydrogel matrix, the mixture-A hydrogel containing Cy5.5-Her was hypodermically injected into SK-BR-3 tumor-bearing nude mice. A single injection of the Cy5.5-Her-loaded hydrogel maintained a higher fluorescence intensity at the injection site than the weekly injection of the Cy5.5-Her solution during the 4-week examination period (Figure 6A-B), indicating the slow and constant release of Herceptin from the hydrogel depot. As a targeted antibody, Herceptin can actively accumulate into the HER2+ breast tumors 7, 8. Compared to the weekly pulsed injections of the Herceptin solution, the sustained release of Herceptin from the hydrogel depot adjacent to the tumor tissue led to a higher accumulation of the antibody in the tumors and less distribution in the major organs (Figure 6C-D).

The *in vivo* anticancer experiments demonstrated that a single injection of the 25 mg/kg Herceptin-loaded mixture-A hydrogel more effectively suppressed tumor growth than the weekly pulsed injections of the Herceptin solution under the same total dosage of the antibody (Figure 7B-C). The best inhibition effect on tumor growth was achieved by a single administration of the 50 mg/kg Herceptin-loaded hydrogel formulation. These findings also indicated that the released Herceptin maintained high bioactivity and did not suffer from obvious denaturation or degradation in the gel depot. Furthermore, the body weights and histological observations did not indicate any anomalies in any of the groups treated with Herceptin (Figure 7E and Figure 8C).

Immunohistochemical analysis further verified that the sustained delivery of Herceptin induced an immune response, which included the promotion of Akt dephosphorylation and activation of an ADCC response (Figure 9). The ADCC response is positively correlated with the antibody concentration in the tumors. The higher antibody aggregation in the tumors, the stronger the ADCC response. However, echocardiography revealed that multiple injections of 6.25 mg/kg Herceptin solution and a single administration of the 50 mg/kg Herceptin-loaded hydrogel formulation resulted in a marked decrease in the LVEF (Figure 8B). In contrast, the LVEF of mice that were treated with the 25 mg/kg Herceptin-loaded hydrogel exhibited no difference from the control group. These findings suggested that compared to the weekly pulsed administration of the Herceptin solution, the sustained release of an appropriate concentration of Herceptin enhanced the anticancer efficacy and reduced the administration frequency without causing drug-induced cardiotoxicity.

Finally, a HER2+ recurrent breast tumor model was established by imitative BCT on nude mice. We found that the anti-relapse efficacy was dependent on the dosage of Herceptin (Figure 10B-C). Both the Herceptin solution and Herceptin-loaded hydrogel treatments at the same dosage of the drug (25 mg/kg Herceptin) successfully suppressed tumor relapse, but the weekly pulsed injection of the Herceptin solution resulted in an obvious reduction in the LVEF (Figure 11B). In contrast, no obvious cardiotoxicity was detected in the mice treated with the hydrogel formulation. This outcome was consistent with the *in vivo* anticancer experiments. Notably, the injection site of the hydrogel formulation was 5 mm away from the excision site of the tumor to allow for the convenient observation and measurement of the recurrent tumors in the present study. In the practice use, the hydrogel carrier can be facilely employed as a temporary filling material to fill irregular defects and improve the cosmetic effects of breasts after BCT. Meanwhile, since the released antibody is closer to the lesion, the anti-relapse efficacy of Herceptin maybe be further increased by injection of the hydrogel formulation into the surgical site after BCT.

The cardiotoxicity of Herceptin is a crucial concern. Although the definite mechanism of Herceptin cardiotoxicity has not been completely determined, it is generally believed that the cardiac dysfunction induced by Herceptin is related to increased myocardial oxidative/nitrative stress and apoptosis, as well as changes in the expression of myocardial genes that are essential for cardiac and mitochondrial functions, adaptation to stress, and DNA repair 64. Recently, Tzahor found that although HER2 is not overexpressed in cardiomyocytes, HER2 plays an important role in cardiomyocyte proliferation and regeneration 69. Epstein et al. revealed that HER2 could affect the formation of a functional receptor for the vascular guidance molecule semaphorin 3d (Sema3d), and they speculated that inhibition of HER2 in endothelial cells may lead to endothelial dysfunction and secondary myocardial dysfunction 70. According to our outcomes, the administration of the 25 mg/kg Herceptin-loaded mixture-A hydrogel did not cause any cardiotoxicity (Figure 8B and Figure 11B). This characteristic was owed to the sustained release of an appropriate concentration of Herceptin and a subsequently higher accumulation of the antibody in the tumors and less distribution of the antibody in the major organs (Figure 6C-D). Consequently, this Herceptin-loaded hydrogel, which increases therapeutic efficacy and improves patient compliance while significantly lowering cardiotoxicity, has great potential for preventing the local relapse of HER2+ breast tumors following BCT.

## Conclusion

In this study, we successfully developed a localized and long-acting Herceptin delivery system to prevent the local relapse of HER2+ breast tumors after BCT. Injectable and thermosensitive PLGA-PEG-PLGA mixture hydrogels were fabricated based on a practical blending approach and were then employed to deliver Herceptin. The gel performance, *in vitro* and *in vivo* gel persistence and drug release profiles of mixture hydrogels were facilely modulated by simply changing the mix proportion. The Herceptin-loaded mixture-A hydrogel displayed a sustained drug release pattern *in vitro* for up to 80 days, which is the longest period Herceptin delivery that has ever been reported. The *in vivo* biodistribution studies showed that compared to the weekly injections of Herceptin solution for 4 weeks, a single hypodermic injection of this hydrogel system adjacent to the tumor site increased the intratumoral antibody accumulation, leading to significantly enhanced *in vivo* antitumor efficacy. In a locally recurring HER2+ breast tumor nude mouse model, a single administration of the Herceptin-loaded hydrogel as well as the weekly injection of the Herceptin solution successfully inhibited tumor relapse. Furthermore, both the antitumor and anti-relapse experiments demonstrated that the weekly pulsed injection of the Herceptin solution resulted in a marked reduction in the LVEF; however, the slow and steady release of Herceptin from the hydrogel depot effectively prevented cardiotoxicity. These results suggested that this hydrogel system efficiently enhances the therapeutic efficacy, reduces the administration frequency and lowers the systemic side effects in the treatment of HER2+ breast tumors. In future clinical practice, the administration of Herceptin-loaded hydrogels at the surgery site after BCT has great potential for preventing the local relapse of HER2+ breast tumors, as well as provides a temporary filling material to offer better cosmetic effects and patient compliance.

## Methods

### Materials

Herceptin was purchased from Roche Pharma (Schweiz) Ltd. PEG (MW =1000 and 1500) and Sn(Oct)_2_ were obtained from Sigma-Aldrich. LA and GA were purchased from Hangzhou Medzone Biotech Ltd. (China). Cyanine5.5 N-hydroxysuccinimide ester (Cy5.5-NHS) was obtained from Lumiprobe. An anti-CD69 polyclonal antibody was obtained from Bioss Antibodies Ltd. (USA), and an AKT-phospho-S473 mouse monoclonal antibody was purchased from Proteintech Group, Inc. Other reagents were obtained from Sinopharm Chemical Reagent Co. and directly used.

### Animals

Female ICR mice weighing approximately 20 g and female BALB/c nude mice weighing approximately 18 g were obtained from SLAC Laboratory Animal Co., Ltd. (Shanghai, China). The nude mice were raised under pathogen-free conditions. All animals were raised in cages under a controlled temperature of 22-25 °C and kept on a 12 h light-dark cycle. The mice were given free access to standard laboratory feed and tap water. All *in vivo* experiments were conducted in conformity to the “Principles of Laboratory Animal Care” (NIH publication #85-23, revised 1985) and were approved by the Ethics Committee of Fudan University.

### Synthesis and characterization of PLGA-PEG-PLGA triblock copolymers

Two PLGA-PEG-PLGA triblock copolymers were synthesized by ring-opening copolymerization of LA and GA in the presence of PEG as the macroinitiator using Sn(Oct)_2_ as the catalyst 59, 71. Considering copolymer-1 as an example, 20.0 g PEG1000 was transferred into a three-necked flask and then dried under vacuum with mechanical agitation at 130 °C for 3 h. Next, 46.6 g LA and 9.4 g GA were added into the three-necked flask followed by vacuum drying for 15 min to remove moisture in the monomers. Then, an Sn(Oct)_2_ solution in toluene was dropped into the flask, and the toluene was removed by decompression. The reaction occurred for 12 h at 150 °C in the argon protection environment. After completion of the polymerization reaction, the crude products were decompressed at 120 °C for 3 h and then washed with water at a temperature of 80 °C 3 times to eliminate the residual monomers and low MW products. The residual water was removed by lyophilization, and the obtained products were collected and stored in a refrigerator at -20 ºC. Copolymer-2 was synthesized via a similar process.

To confirm the chemical structures and compositions of the triblock copolymers, a 400 MHz ^1^H-NMR (AVANCE III HD, Bruker) spectrometer was used to record the ^1^H-NMR spectra in the presence of CDCl_3_ as a solvent and tetramethylsilane (TMS) as an internal standard. A GPC system (Agilent 1260) was used to determine the MWs and molar mass dispersity (*Đ*_M_s) values of the samples with tetrahydrofuran as an eluent at a flow rate of 1.0 mL/min at 35 °C. A series of polystyrene samples with narrow distributions were used as standards to calculate the MWs. Mixtures A, B, and C were obtained by mixing copolymer-1 and copolymer-2 with weight mix proportions of 7:3, 5:5 and 3:7, respectively.

### Phase diagram

The vial-inverting approach 59 with a 1 °C per step heating rate was used to determine the phase diagram. Briefly, a polymer mixture with a fixed mix proportion was dissolved in NS to form aqueous solutions containing different amounts of polymers. Then, 0.5 mL of the polymeric solution was added into 2-mL vials, and the vials containing the specimens were immersed in a water bath. At each water bath temperature, the specimens were equilibrated for 15 min. If no visible flow was observed when inverting the vials 180° for 30 s, the specimen was thought to be a gel. The temperature range for the measurements was from 10 to 60 ºC.

### Dynamic rheological analysis

The rheological properties of the aqueous polymer solutions with rising of temperature were analyzed by a dynamic stress-controlled rheometer (Kinexus, Malvern) equipped with a cone plate (diameter: 60 mm, cone angle: 1°, gap: 0.03 mm). Low-viscous silicon oil was used to overlay the margin of the plate to prevent water evaporation from the samples. Temperature sweep experiments were performed with a heating rate of 0.5 °C/min from 10 to 45 °C, which was managed via a Peltier temperature controller, and the frequency was set at 1.59 Hz.

### *In vitro* Herceptin release

The mixtures with different weight mix proportions of copolymer-1 and copolymer-2 were dissolved in NS to obtain 25 wt% aqueous polymer solutions. A predetermined amount of Herceptin was added into the aqueous polymer solutions, and 0.5 mL of the Herceptin-loaded polymer solution was then transferred into 15-mL test tubes (with an inner diameter 15 mm). After incubating the test tubes containing the samples in a water bath at 37 ºC for 5 min, the aqueous polymer solutions containing Herceptin turned into *in situ* hydrogels, and 10 mL of pre-warmed PBS containing 0.025% NaN_3_ was transferred into the test tube as the release medium. The samples were shaken at 50 rpm for the entire period of the examination. 5 mL of the release medium was removed at designated time intervals and replaced by the same volume of fresh buffer immediately to maintain the sink condition. The amount of Herceptin in the release medium was quantified by a Micro BCA Protein Assay Kit (Thermo Scientific, USA), and the release medium of the drug-free hydrogel was also taken out at the same time point as the control to deduct the effect of degradation products. In addition, optical photographs of the Herceptin-loaded hydrogel systems were also acquired at the indicated time points to evaluate the *in vitro* degradation of the different mixture hydrogels.

### *In vivo* hydrogel degradation

The aqueous solutions of copolymer mixtures were sterilized by Co-60 before use. Intraperitoneal injection of 4% chloral hydrate solution was performed to anesthetize female ICR mice, and then 0.1 mL mixture aqueous solutions were hypodermically injected into the dorsal areas of the mice. Some mice were sacrificed and dissected at predetermined time points, and the remaining hydrogels were photographed using a digital camera.

### *In vivo* biodistribution of Herceptin

To noninvasively monitor the *in vivo* release of Herceptin from the hydrogel matrix, Cy5.5, which is a fluorescence probe, was used to label Herceptin. First, Herceptin was dialyzed in a Float-A-Lyzer system (Spectra/Por, USA, MWCO 100 kDa) to remove excipients and was then lyophilized. Then, the purified antibody was reacted with Cy5.5-NHS at a molar ratio of 1:4 at pH 8.4 for 12 h, and the unreacted probe was then eliminated by dialysis in a dialysis bag (MWCO 3500 Da) for 12 h. Finally, Cy5.5-Her was obtained after lyophilization.

SK-BR-3 tumor cells (2×10^6^ cells), which are human HER2+ breast tumor cells, were suspended in 0.1 mL DMEM (Gibco) and inoculated hypodermically into the first right breast fat pad of female BALB/c nude mice to develop tumors. 14 days later, the mice were randomly divided into the following 4 groups (*n* = 4): (A) a single hypodermic injection of 25 mg/kg (containing 0.5 mg/kg Cy5.5-Her) Herceptin-loaded mixture-A hydrogel, (B) weekly hypodermic injections of 6.25 mg/kg (containing 0.125 mg/kg Cy5.5-Her) Herceptin solution for 4 weeks, (C) a single hypodermic injection of 25 mg/kg (containing 0.125 mg/kg Cy5.5-Her) Herceptin-loaded mixture-A hydrogel, and (D) a single hypodermic injection of 6.25 mg/kg (containing 0.125 mg/kg Cy5.5-Her) Herceptin solution. The polymer concentration of mixture-A was 25 wt%, the drug loading amount was 5 mg/mL, and the injection volume of the hydrogel was 100 µL. The injection site was 5 mm away from the tumor, and isoflurane was used as the anesthetic. For groups A and B, the amount of Cy5.5-Her in the Herceptin-loaded hydrogel was 4-fold greater than that of the Herceptin solution. However, the Herceptin solution containing Cy5.5-Her was injected into the nude mice once per week for four weeks. Hence, the total amount of the fluorescent molecule was equal in group A and group B. Fluorescence imaging was performed at 0, 1, 2, 4, 7, 14, 21, and 28 days post-administration using an optical and X-ray small animal imaging system (In-Vivo Xtreme, Bruker) with 690 nm as excitation wavelength and 790 nm as emission wavelength. On day 28 post-administration, all the nude mice were euthanized, and their tumors and major organs were dissected and further observed by fluorescence imaging. For groups C and D, the same amount of Cy5.5-Her was added into the Herceptin-loaded hydrogel formulation and Herceptin solution. Fluorescence imaging was performed at 0, 1, 2, 4, and 7 days post-injection.

### *In vivo* antitumor efficacy

BALB/c nude mice bearing human SK-BR-3 cancer xenografts were established by the injection of a suspension of SK-BR-3 tumor cells (2 x 10^6^ cells). As the tumor volume reached approximately 50 mm^3^, the SK-BR-3 breast tumor-bearing nude mice were randomly divided into 4 groups (*n* = 5) and treated with (A) NS (one hypodermic injection of 100 µL NS), (B) weekly hypodermic injections of 6.25 mg/kg Herceptin solution for 4 weeks, (C) a single hypodermic injection of the 25 mg/kg Herceptin-loaded mixture-A hydrogel, (D) a single hypodermic injection of the 50 mg/kg Herceptin-loaded mixture-A hydrogel. The polymer concentration of mixture-A was 25 wt%, the drug loading amount was 5 or 10 mg/mL, and the injection volume of the hydrogel was 100 µL. The tumor volume was tested twice per week and calculated by the following equation: 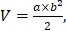 where a and b are the longest and shortest diameters of the tumors, respectively. The body weights of the mice were weighed twice per week as an indicator of systemic toxicity.

### *In vivo* anti-relapse efficacy

The relapse mouse model was established based on the SK-BR-3 breast tumor-bearing nude mice. First, BALB/c nude mice received a hypodermic injection of a suspension of SK-BR-3 tumor cells to develop tumors. When the tumor volume was ∼100 mm^3^ after 21 days of tumor growth, the mice were deeply anesthetized and fixed on a surgery board. The epidermis around the tumor was disinfected by iodine and medicinal alcohol. Then, a surgical towel with a small window was placed on the nude mice. A lumpectomy was performed to imitate the clinical breast-conserving surgery. Then, approximately 1 mm^3^ of the tumor mass was separated from the original tumor and then placed into the excision site to imitate the residual tumor after the breast-conserving surgery. Finally, the surgical wound was sutured with 6.0 Monocryl sutures (Ethicon, USA), reinforced with medical glue and disinfected with iodine.

To ensure that the strength of the wound was sufficiently strong to tolerate the injection pressure, different treatments were performed at one day post-surgery, and the mice were randomly divided into the following 5 groups (*n* = 5): (A) NS (one hypodermic injection of 100 µL NS), (B) weekly hypodermic injections of 6.25 mg/kg Herceptin solution for 4 weeks, (C) a single hypodermic injection of the 2.5 mg/kg Herceptin-loaded mixture-A hydrogel, (D) a single hypodermic injection of the 12.5 mg/kg Herceptin-loaded mixture-A hydrogel, and (E) a single hypodermic injection of the 25 mg/kg Herceptin-loaded mixture-A hydrogel. The polymer concentration of mixture-A was 25 wt%, the drug loading amount was 0.5, 2.5 or 5 mg/mL, and the injection volume of the hydrogel was 100 µL. The injection site was 5 mm away from the center of the sutures. The tumor size was measured twice per week except on the first week due to the occurrence of postoperative complications, such as edema. The body weights of the mice were also weighed twice per week as an indicator of systemic toxicity.

### *In vivo* LVEF measurement and histological analysis

On day 28 post-treatment, all the nude mice in both the antitumor and anti-relapse experiments were anesthetized by isoflurane. The cardiac functions of the mice were analyzed by a Vevo LAZR system (FujiFilm VisualSonics Inc., USA), and LVEF values were then calculated according to eq 1 as follows:


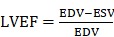
 (1)

where EDV and ESV are the end-diastolic volume and end-systolic volume, respectively. EDV and ESV were calculated by the Teichholz equation (eq 2) as follows:


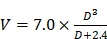
 (2)

where *D* is the left ventricular diameter 62, 63. Subsequently, all the nude mice were sacrificed, and the tumors were dissected and weighed. The major organs of the mice were also harvested and fixed in 4% paraformaldehyde solution. The tumor tissues and various organs were sectioned, and H&E staining and TUNEL assays were then performed according to the manufacturer's protocol. Immunohistochemical staining of pAkt and CD69 was also performed on the tumor tissues according to the manufacturer's protocol to further demonstrate the antibody efficacy and immune response. Finally, the obtained sections were stained with DAB (Maixin Biotech, China) and counterstained with hematoxylin. Micrographs of the H&E-, CD-69- and pAkt-stained samples were acquired under an inverted microscope (Eclipse LV100ND, Nikon), and the micrographs of the TUNEL-stained samples were acquired under another inverted fluorescence microscope (Eclipse Ti-SR, Nikon).

### Statistical analysis

One-way analysis of variance was employed to evaluate the significant difference between the two groups. *p* < 0.05 was considered statistically significant.

## Supplementary Material

Supplementary figures.

## Figures and Tables

**Table 1:** List of the triblock copolymers and their indicated mixtures in the present work

Sample	Mix proportion^c)^	*M*_n_^ d)^	LA/GA (mol/mol)^d)^	*M*_n_^e)^	*Đ*_M_^e)^
Copolymer-1^a)^		1505-1000-1505	4	5590	1.26
Copolymer-2^ b)^		1250-1500-1250	4	5550	1.14
Mixture-A	7:3	/	4	5570	1.22
Mixture-B	5:5	/	4	5560	1.19
Mixture-C	3:7	/	4	5560	1.16

a) Copolymer-1 was not dissolved in water due to its strong hydrophobicity. b) Copolymer-2 easily formed a sol in water. c) The weight mix proportion of copolymer-1 and copolymer-2. d) The PEG block's *M*_n_ was given by Aldrich. The *M*_n_ of the PLGA blocks and the molar proportion of LA/GA were obtained from ^1^H NMR spectra. e) Determined by GPC.

## Abbreviations

ADCC: activate antibody-dependent cellular cytotoxicity
BCT: breast-conserving therapy
Cy5.5-Her: Cy5.5-labeled Herceptin
Cy5.5-NHS: Cyanine5.5 N-hydroxysuccinimide ester
*Đ*_M_: molar mass dispersity
EMA: European Medicines Agency
FDA: U.S. Food and Drug Administration
*G′*: storage modulus
*G*′′: loss modulus
GA: glycolide
GPC: gel permeation chromatography
H&E: hematoxylin/eosin
LA: D,L-lactide
LVEF: left ventricular ejection fraction
MAPK: MAP kinase
MW: molecular weight
NK: natural killer
NMR: nuclear magnetic resonance
NS: normal saline
PBS: phosphate-buffered saline
PI3K: PI3 kinase
PLGA-PEG-PLGA: poly(lactic acid-*co*-glycolic acid)-*b*-poly(ethylene glycol)-*b*-poly(lactic acid-*co*-glycolic acid)
rHuPH20: recombinant human hyaluronidase
Sema3d: semaphorin 3d
*T*_g_: sol-gel transition temperature
TMS: tetramethylsilane
TUNEL: terminal deoxy-nucleotidyl transferase-mediated dUTP-biotin nick end labeling
