# Supplementary material for: Injectable hydrogels for the sustained delivery of a HER2-targeted antibody for preventing local relapse of HER2+ breast cancer after breast-conserving surgery
Source: Theranostics. 2019 Aug 14;9(21):6080–98. doi: 10.7150/thno.36514 (PMC6735507; doi:10.7150/thno.36514)
Supplement: Supplementary file 1 — Supplementary figures. [file thnov09p6080s1.pdf]

Supporting information

Injectable hydrogels for the sustained delivery of a HER2-targeted antibody for preventing local relapse of HER2+ breast cancer after breast-conserving surgery

Xiaobin Chen[1,†] , Maoli Wang[2, †], Xiaowei Yang[1], Yaoben Wang[1], Lin Yu[1, *], Jian Sun[2,*] and Jiandong Ding[1]

1. State Key Laboratory of Molecular Engineering of Polymers, Department of Macromolecular Science, Fudan University, Shanghai 200438, China

2. Department of Breast Surgery, Obstetrics and Gynecology Hospital, Fudan University, Shanghai 200011, China

[†] Authors contributed equally.

[*] Corresponding authors: yu_lin@fudan.edu.cn and surgian@163.com

Keywords: Injectable hydrogel; Breast-conserving surgery; Herceptin; Cardiotoxicity; Anti-relapse; Immunotherapy

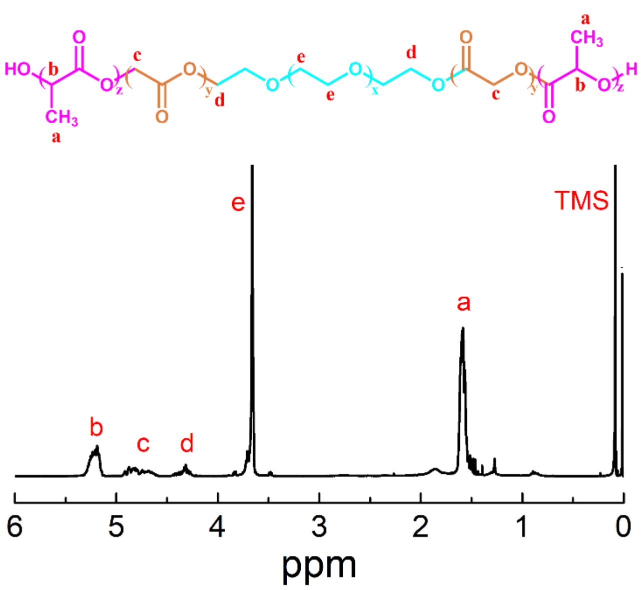

Figure S1. ^{1}H NMR spectrum of a PLGA-PEG-PLGA triblock copolymer (copolymer-2) in $CDCl_3$.

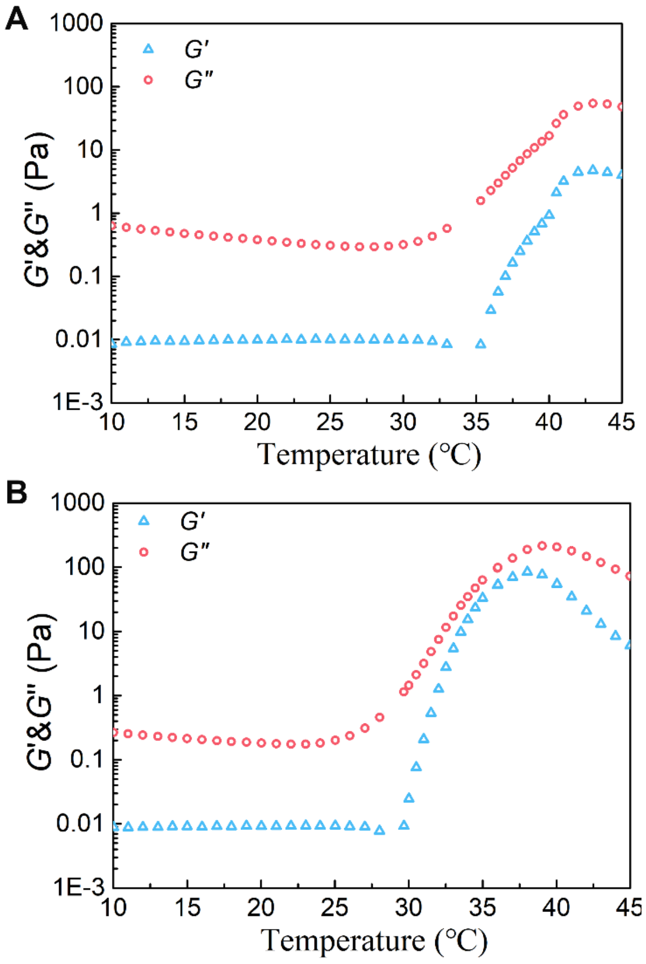

Figure S2. The storage modulus (G') and loss modulus (G'') of the aqueous solutions of (A) copolymer-2 and (B) mixture-C as a function of temperature. Shear frequency: 1.59 Hz; heating rate: 0.5 °C/min. The polymer concentration was 25 wt%.

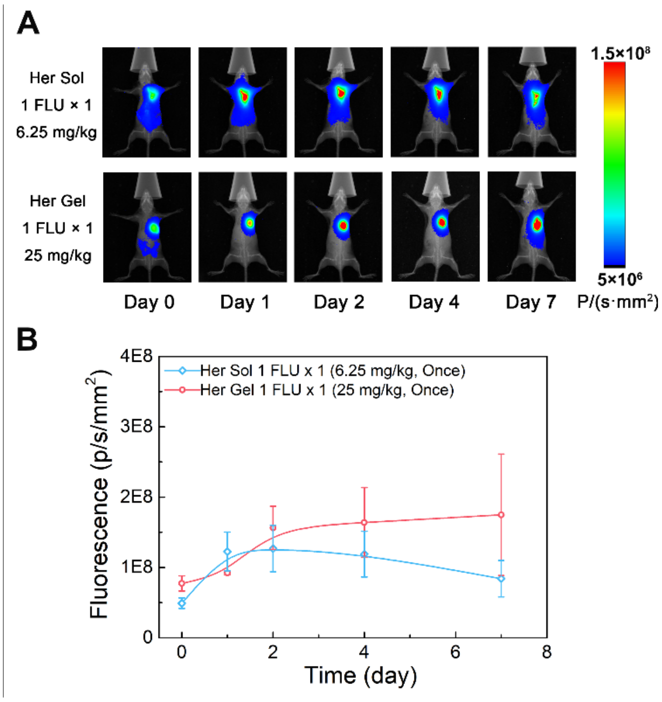

Figure S3. (A) Real-time *in vivo* fluorescent imaging of SK-BR-3 tumor-bearing nude mice with subcutaneous injection of Cy5.5-Her solution or the Cy5.5-Her-loaded mixture-A hydrogel. The fluorescence intensity of Cy5.5-Her in the hydrogel was equal to that of Cy5.5-Her solution. All the images shown in the same group are from the same mouse. (B) Semi-quantitative analysis of the fluorescence signals at the injection sites as a function of time.
